# Patient and public involvement in health technology decision-making processes in Brazil

**DOI:** 10.11606/s1518-8787.2020054002453

**Published:** 2020-11-27

**Authors:** Ana Carolina de Freitas Lopes, Hillegonda Maria Dutilh Novaes, Patricia Coelho de Soárez

**Affiliations:** I Universidade de São Paulo Faculdade de Medicina Programa de Pós-Graduação em Saúde Coletiva São PauloSP Brasil Universidade de São Paulo. Faculdade de Medicina. Programa de Pós-Graduação em Saúde Coletiva. São Paulo, SP, Brasil; II Universidade de São Paulo Faculdade de Medicina Departamento de Medicina Preventiva São PauloSP Brasil Universidade de São Paulo. Faculdade de Medicina. Departamento de Medicina Preventiva. São Paulo, SP, Brasil

**Keywords:** Community Participation, Social Participation, Stakeholder Participation, Technology Assessment, Biomedical, Biomedical Technology, Brazilian Health Surveillance Agency, Supplemental Health

## Abstract

**OBJECTIVE:**

The study aims to characterize and discuss the processes of patient and public involvement (PPI) in the Brazilian Health Regulatory Agency (Anvisa), the National Committee for Health Technology Incorporation (Conitec), and the National Agency for Supplementary Health (ANS) in Brazil.

**METHODS:**

This is an exploratory, descriptive, and comparative study, conducted by analyzing the public documents and regulation of the three institutions.

**RESULTS:**

The mechanisms for PPI included public consultations, public hearings, participation in advisory committees, and health technology evaluation requests. Anvisa conducted 187 public consultations between 1999 and 2018, gathering 10,699 contributions. In total, 76 (41%) public consultations did not present information about the contributions received. Conitec carried out 234 public consultations and received 53,174 contributions between 2011 and 2018. It was identified that 70 (23%) recommendations from Conitec did not go through public consultation, and 26 (8%) recommendations changed after public consultation. Recommendation changes seemed to have occurred especially in cases with a greater number of contributions in the public consultation process. ANS conducted eight public consultations regarding the list of health procedures and events covered by health insurances between 2000 and 2018, and it received 31,498 contributions. For three public consultations, there was no information about the number of contributions received.

**CONCLUSIONS:**

There are regulatory advances and institutional activity supporting PPI in highly technical decision-making processes in Brazil, although heterogeneously among the analyzed institutions. The power of PPI to influence health technology deliberative processes still requires in-depth studies, including the characterization of stakeholders and the legitimacy of decisions.

## INTRODUCTION

The institutionality of patient and public involvement (PPI) – as well as the very construction of democracy – is associated with specific historical contexts^1,2^. Especially in Latin America, where political instability and structural inequalities are persistent, it has been challenging to propose and to maintain policies that ensure the participation of society in decision making processes^1,2^.

Community participation in health-related decisions has been included as a constitutional rule since the Brazilian Unified Health System (SUS) was created and has been regulated through as National Health Conferences and Health Councils^3^. In the following years, PPI was a subject of debate and political action, and other mechanisms for engagement in health decision-making were structured ^6^.

At the same time, the *Política Nacional de Gestão de Tecnologias em Saúde* (PNGTS—National Policy on Health Technologies Management), established the use of technical and scientific knowledge in health decisions, even though it was based on the principle of public engagement^7^. In this document, the management of health technologies is defined as the processes of evaluation, incorporation, dissemination, use, and exclusion of technologies in the health system^7^. Therefore, it comprises regulatory approval of medicines, health care products, and other procedures related to health, as well as the decisions related to health insurance plans and incorporation of technologies in SUS. At the national level, the management of health technologies is carried out by the Brazilian Health Regulatory Agency (Anvisa), the National Committee for Health Technology Incorporation (Conitec) and the National Agency of Supplementary Health (ANS).

Anvisa’s activities include the regulatory approval of health technologies, from clinical trials to marketing authorization, with scientific proof of safety and efficacy^8^. Additional analyses on safety, efficacy, accuracy, and effectiveness, as well as comparative economic evaluation of benefits and costs with technologies already in the market are required in order to authorize the production and commercialization of a technology before its offer at SUS or its inclusion in the minimum list of supplemental health procedures and events^4,9^. The decision to incorporate, to exclude or to change technologies in SUS is the responsibility of the Brazilian Ministry of Health (MH), advised by Conitec^4^. In supplementary health, ANS Board of Directors is responsible for the aforementioned decision, after consulting the *Comitê Permanente de Regulação da Atenção à Saúde* (COSAÚDE—Permanent Healthcare Regulation Committee)^9^.

The institutions responsible for the management of health technologies must reconcile technical decisions and the social participation, following the Guidelines of SUS and the current regulation. Initiatives of PPI carried out by Anvisa, Conitec, and ANS were the subject of individual analyses in other studies^10^, but not yet comparatively based on the scope of health technology management, as described in PNGTS. This work aims to characterize and to discuss the mechanisms of patient and public involvement in the institutions responsible for the management of health technologies at the national level in Brazil.

## METHODS

This is an exploratory, descriptive, and comparative study of the formal processes of patient and public involvement planned and implemented by Anvisa, Conitec, and ANS. For this purpose, documentary analysis was conducted as a method of data collection, consulting official documents and specific regulation. The primary source of information was the websites of the institutions, understood as the main tool for information access available to society.

Documents published in the period between the legal creation of the institution and December 2018 were included. Thus, for Anvisa, the period considered was between 1999 and 2018; between 2000 and 2018 for ANS; and for Conitec, between 2011 and 2018. Incomplete or absent information was formally requested from the institutions with the channels provided for the Brazilian freedom of information law (Law No. 12,527/2011)^15^.

Descriptive analysis was used for data analysis. Information about the mechanisms of PPI formally planned and public consultations already carried out regarding health technologies were detailed for each institution. The presentation of public consultation was favored—among other spaces—by the configuration of open participation, with direct communication between individuals and institutions, without the need for representatives^16^. Moreover, its realization is always prior to decision-making, with the prerogative of being able to amend it.

According to PNGTS, “medicines, equipment and technical procedures, organizational, informational, educational and support systems, and care programs and guidelines through which health care are provided to the population” are considered as health technologies^7^.

The mechanisms of PPI were identified as processes used by the society to express its demands. All individuals or legal entities that are not part of the governmental structure were considered as society, including those who access SUS and health insurance plans, institutions and health professionals, industry, and service sectors, as well as civil society organizations.

The processes of PPI were classified into five categories: health technology evaluation requests, participation in advisory committees, public consultations, public hearings, and ombudsman’s offices. Acting health technology evaluation requests is the society’s prerogative to initiate the process related to the management of technologies. It refers to the application for registration in Anvisa, incorporation in the SUS through Conitec or inclusion in ANS list.

Advisory committees refers to the participation of social representatives in collective instances, created by the public authorities’ initiative, like Health Councils. Consultations and public hearings are mechanisms in which any stakeholder can contribute, both in writing or in person. Institutional public ombudsman’s offices are instances of receipt, referral, and resolution of complaints, requests, and information related to public policies and services, which are open to any citizen^16^.

Public consultations carried out by each institution were categorized by theme and detailed regarding the deadline for sending contributions and number of contributions received. Public consultations carried out by areas of Anvisa related to medicines, health products, and food were included, because they fit the concept of health technology presented in PNGTS. Public consultations on pesticides, cosmetics, general sanitizer, tobacco, blood, tissues and organs, and internal management were excluded, because they dealt with topics other than technologies used in the direct provision of health care. Regarding inclusion, the deadline for submitting contributions should be until December 31, 2018.

For Conitec, public consultations on medicines, products or procedures were included, with processes closed until 2018. The date of publication of the decision by the MH was considered as the end of the process. The information about the contributions was obtained with the final version of the technical reports available on Conitec’s website. Public consultations on *Protocolos Clínicos e Diretrizes Terapêuticas* (CPTG—Clinical Protocols and Therapeutic Guidelines) were excluded, since they refer to documents guiding clinical conduct, services organization and implementation of technologies incorporated in previous decisions. The process of developing CPTG—including the mechanisms of PPI—deserves a thorough analysis^14,17^.

For ANS, it were included public consultations on the update of procedures and events covered by health insurances, therefore directly related to health technologies, held until 2018. Public consultations on topics unrelated to the management of health technologies were excluded, such as the financial management issues of health insurance plan or document standardization.

This work composes the research project “Social participation in the incorporation of technologies into SUS,” approved by the Research Ethics Committee of the Medical School of the Universidade de São Paulo. The documents used in this study are publicly accessible.

## RESULTS

In total, 606 documents on the legal framework of the institutions and documents of processes conducted by them were analyzed. Out of these, the following were included: 187 publications about public consultation and 99 social participation analysis reports made available by Anvisa; 304 technical reports published by Conitec; eight opening terms of public consultations and five public consultations reports published by ANS; in addition to the laws of creation of each institution (n = 3). The formal mechanisms of PPI identified in the institutions will be presented in a comparative way, followed by the details of the public consultations held.

### Formal mechanisms of Patient and Public Involvement

The Box presents the formal mechanisms of PPI by institution. Only the producer of a certain technology can request from Anvisa the authorization to conduct clinical research, to produce, and to market new products. It is also up to them to demand the price specification of medicines and post-registration changes. Anvisa decisions on these issues are exclusively based on technical and procedural aspects, without formal opening to external participation, in addition to the plaintiff itself.

**Box:** Formal mechanisms of PPI in the management of health technologies at the federal level in Brazil, by institution.

Institution	Health technology evaluation requests	Committees	Public consultation	Public hearing	Ombudsman’s office
Anvisa	Producer of technology	**Permanent advisory board** Advisory and proposal-related character Representatives of society (number of nominees): BCI (1), BTC (1), scientific community (2), consumer protection (2), BHC (1) and CNSaúde (1) **Sectoral chambers** Advisory character Composition and duration fixed on a case-by-case basis It must include government, industry, and civil society	Optional	Optional	Has its own
Conitec	Anyone (government, industry or civil society)	**Full court of Conitec** Permanent and deliberative Representatives of society (number of nominees): BHC (1) and BFCM (1)	Mandatory	Optional	Submitted to the Ombudsman’s office of the Brazilian Ministry of Health
ANS	Anyone (government, industry or civil society)	**Permanent Committee for Health Care Regulation (Cosaúde)** Permanent and advisory Representatives of society (number of nominees): BHC (1), health professionals (4), health service providers (3), industry (2), labor unions (3), supplemental health companies (6), consumer protection (4), patients (2) **Technical chambers** Thematic discussions Composition and duration fixed on a case-by-case basis	Optional	Optional Mandatory only in case of drafting of a law in the scope of ANS	Has its own

Anvisa: Brazilian Health Regulatory Agency; Conitec: National Committee for Health Technology Incorporation into the SUS; ANS National Agency of Supplemental Health; BHC: Brazilian Health Council; CNSaúde: National Confederation of Health, Hospitals, Establishments, and Services; BFCM: Federal Council of Medicine; BCI: Brazilian Confederation of Industry; BTC: Brazilian Trade Confederation.

The participation in advisory committees stems from Anvisa’s permanent advisory board and in temporary thematic sectoral chambers, both of advisory nature. Out of the 13 members of the permanent advisory board, eight are representatives of institutions interested in the agency’s regulation, and the others are federal, state, and municipal managers. It is expected that Anvisa conduct consultations and public hearings, but without mandatory aspect. Anvisa also has its own ombudsman’s office with legally defined competencies.

According to the regulation, any interested party may act as a plaintiff with Conitec and ANS aiming to the incorporation of their interests into the SUS or in supplemental health. The restrictions are technical and procedural in nature. Among the requirements, the systematized presentation of scientific evidence on clinical aspects of the technology and specific studies of economic evaluation and budgetary impact are highlighted.

Conitec’s recommendations regarding the request for incorporation are provided by a plenary, a permanent and deliberative committee. The deliberative character of the plenary refers to the possibility of vote of its members in relation to the final recommendation of the request for incorporation, even though the final decision relies on the MH. Among the 13 members of the plenary, there are representatives of the Brazilian Health Council (BHC) and Brazilian Federal Council of Medicine (BFCM). The other are from the MH itself (including all Departments, Anvisa, and ANS) and state and municipal managers.

Public consultations on the incorporation of technologies in the SUS are mandatory. The received contributions must be examined by the plenary before issuing the final recommendation. Public hearings are expected to be called in cases where they are considered relevant. Conitec does not have its own ombudsman’s office, but it is submitted to the general ombudsman’s office of the Brazilian Ministry of Health, therefore, Conitec is subject to mandatory reply.

The proposals for inclusion in the ANS list are discussed in the COSAÚDE, a permanent and advisory committee. Members include representatives of consumers, operators, health professionals, organized civil society with an interest in the sector, and government entities. consultations and public hearings are optional; in case of drafting of law in the agency’s scope the public hearings are exclusively mandatory. The ANS ombudsman’s office is specific and integrates its own organizational structure.

### Public Consultations

**Figure 1:**
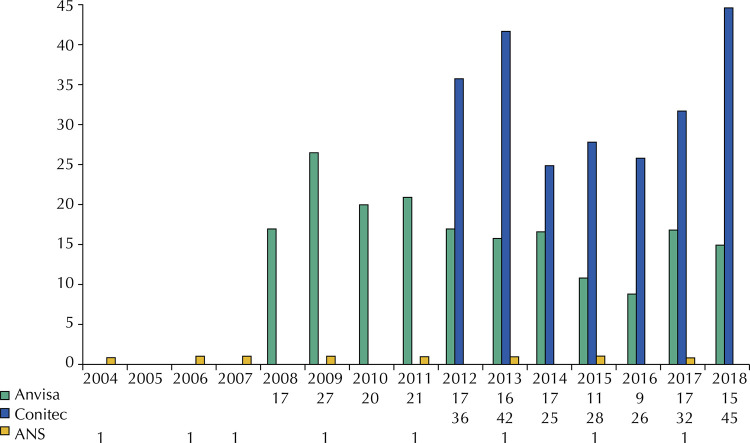
Number of public consultations held per year, for each institution. Anvisa: Brazilian Health Regulatory Agency; Conitec: National Committee for Health Technology Incorporation; ANS: National Agency of Supplemental Health.

Figure 1 shows the temporal comparison between the number of public consultations carried out by Anvisa, Conitec, and ANS about health technologies until 2018.

Anvisa has carried out public consultations on health technologies since 2008, with an average and median of 17 public consultations per year and a total of 187 public consultations conducted between 2008 and 2018.

Conitec conducted an average of 33 public consultations per year between 2011 and 2018 (median = 32), with a total of 234 public consultations in the period. It was observed that 70 Conitec recommendations were issued without public consultation, despite its mandatory character, representing 23% of all recommendations between 2012 and 2018 (supplemental material). Out of these, 58 were recommendations for incorporation and 12 of exclusion (disinvestment), which came from the public sector. In total, 66 (94%) recommendations without public consultation were demanded by the Brazilian Ministry of Health itself. The others had as plaintiff Anvisa, Brazilian Council of Municipal Health Departments (CONASEMS), the Health Department of Pernambuco, and the Judiciary Branch.

ANS started the public consultations on the list of procedures and events in health in 2004, with a biannual frequency, according to the periodicity expected to update the list. Eight public consultations were held between 2004 and 2018.

**Figure 2:**
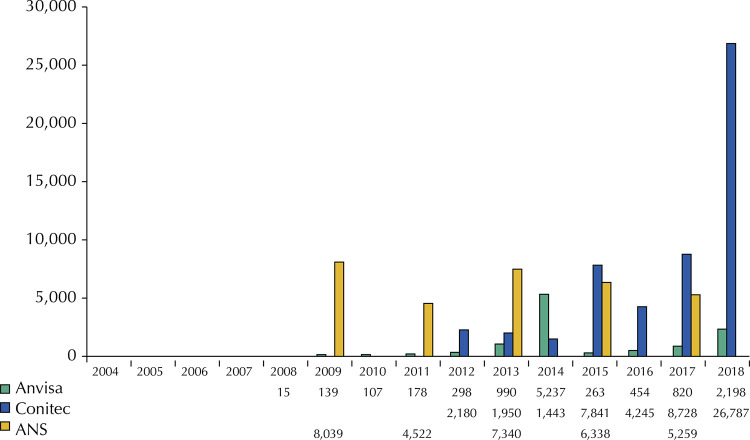
Number of contributions received in public consultations per year, for each institution. Anvisa: Brazilian Health Regulatory Agency; Conitec: National Committee for Health Technology Incorporation into the SUS; ANS National Agency for Supplemental Health.

Figure 2 shows the comparison of contributions received in public consultations.

The volume of contributions received by Anvisa was at least 10,699. A total of 76 (41%) consultations did not have available information on their result, all dated before 2014. The number of contributions per consultation ranged between two and 3,531, with a contribution interval between 10 and 120 days.

Conitec received 53,174 contributions, an average of 227 contributions per public consultation (median = 4,245). The time for contributions ranged between 10 and 47 days. In 11 (5%) public consultations no suggestions were received. Contributions received in 2018 represent half of all contributions already received by Conitec.

ANS received 31,498 contributions in public consultations held on the update of the list between 2009 and 2018. No information was available on the volume of contributions received in the three public consultations conducted before 2009. The average number of contributions received is 2,622 per public consultation (median = 6,338), and the deadline for sending contributions ranged between 12 and 60 days.

Regarding the themes, Anvisa carried out more public consultations about medicines (n = 81; 43%), followed by food (n = 60; 32%) and health care products (n = 46; 25%). Public consultations about food presented the highest volume of contributions (n = 6,274; 59%). Public consultations on medicines received 3,012 contributions (28%), and those on health care products received 1,413 (13%) contributions.

For Conitec, public consultations about medicines were more frequent as well as they received more social inputs: 179 (76%) public consultations and 44,052 (83%) contributions. Procedures were the subject of 35 (15%) consultations and they had 1,870 (4%) contributions. Health care products were the subject of 20 (9%) consultations, with a total of 7,252 (14%) contributions received.

It was identified that 26 (8%) Conitec’s recommendations were changed after the public consultation, mainly from a negative preliminary recommendation to a final recommendation favorable to the incorporation. Most cases (73%) occurred in 2017 and 2018 and they had as plaintiff the company that produces the technology (62% of cases). Note that, out of the 10 public consultations with the highest volume of contributions received by Conitec, four had the preliminary recommendation changed after the public consultation.

## DISCUSSION

This study presented the mechanisms for patient and public involvement in Brazilian institutions responsible for the management of health technologies, at national level, as participation in advisory committees and direct participation of stakeholders by an initial demand or in public consultations, public hearings. Anvisa, Conitec, and ANS totaled 429 public consultations and 95,371 contributions received in the period between the legal creation of the institution and December 2018, indicating the relevance related to public consultations as a mechanism of social participation in these institutions.

On the other hand, it was observed that means of social participation are heterogeneously in each institution. The differences observed in this study are related to the advisory or deliberative nature of the collective bodies, the qualification and quantity of representatives, the binding or discretion of holding public consultations and the provision of information on public consultations held and the effect on the final decisions.

Although included in a larger policy of health technologies management, it was observed that the three institutions have a different scope of action, with different objects of analysis and, consequently, different stakeholders and sources of political and social pressure. This is reflected in the different conceptions of social participation observed among institutions.

The Health Technology Assessment (HTA)–the tool used in the three institutions to perform their evaluations–commonly uses the model of public involvement proposed by Gauvin et al.^18^. The model considers users of the health system, patients, and health professionals as interested in the HTA outcome. This model does not include, at least explicitly, key actors in health technologies management in Brazil: producers of technologies (such as the pharmaceutical industry), health insurance plan operators, and the judiciary branch.

In this study, it was observed that Conitec has a higher frequency of public consultations, due its mandate in health technology incorporation. However, it was identified that, approximately one in four Conitec recommendations, did not go through public consultation, especially when the health incorporation request was made by a governmental body.

The distinct treatment provided by Conitec according to the origin of the demand was identified in previous studies^19,20^. Caetano and colleagues^19^ observed that 32% of Conitec’s recommendations on medicines by June 2016 were made without public consultation, including drugs without registration in Anvisa, all demanded by the MH. Yuba et al.^20^ observed that those demands from the public sector presented a higher frequency of recommendations favorable to incorporation, even though their reports did not present all formal requirements for clinical and economic evidence.

In this work on social participation, the public consultation also received differentiated treatment in government demands, the plaintiff’s proposal was approved without subjecting it to a public consultation, even if they were technologies of relevance to the population covered by SUS. Among the technologies not submitted to public consultations, there was an expressive volume of disinvestment, that is, the withdrawal of technologies that were offered by SUS. Among these cases, some technologies were highly sensitive, in which there is an important organization of civil society, such as people living with HIV and AIDS.

The changes in the recommendation about the incorporation of technologies into SUS after public consultations, especially in cases with a large volume of contributions, may indicate an institutional permeability to the intensity of patient and public involvement, if we consider that the large volume of contributions always implies greater interest of society, which requires, however, analysis of the segments that participated in the contributions. Silva et al.^14^ identified that six Conitec recommendations had the preliminary recommendation amended from “non-incorporation” to “incorporation” between 2012 and 2017. In five of them, a new price was proposed by the plaintiffs or relevant economic fact (availability of generic medicine) between the preliminary recommendation and the final recommendation^14^. These observations highlight the significance of economic and financial dimension in the decisions of technologies incorporation into SUS.

For Anvisa and ANS, the lack of mandatory public participation and the substantial information gap on the processes of social participation in its initial years of operation are highlighted, even after formal request. Other studies have also identified processes conducted by Anvisa^10,12^, Conitec^14,21^, and ANS^13^ aimed to involve society in their decisions. The absence of means of social participation and the advisory nature of the available mechanisms were indicated as significant limitations of an effective social participation in Anvisa and ANS^10^. Regarding Conitec, its recent implementation, lack of systematic and objective methods for analyzing contributions and absence of impact evaluation of the implemented initiatives were highlighted^14,21^.

Mechanisms of social participation are present in countries whose health system and technology management are examples for Brazil^22^. Several methods are used, with the inclusion of representatives in councils and committees^22,24,27,28^. Public consultations were identified in England, Canada (Ontario), Australia, and Germany to receive comments on national clinical reports and guidelines^26,29,30^.

The limitation of this study is the fact that only a quantitative measurement of the social participation in the decision-making arena was carried out, and not an evaluation of the effectiveness of social participation promoted by these organizations. Thus, the evaluation of how managers include the social contributions in their decision-making processes, if they do so, based on the demands of which interested parties and whether they promote equitable social participation is a significant aspect to be observed.

Another significant limitation of this is study is the absence of participants’ identification in these participatory venues. Companies, professionals, consumers, social movements, and isolated individuals were equal for this work, based on the definition of society as any individual or legal entity not part of the governmental structure. As regulatory institutions in a sector with large market interests, it would be mandatory to distinguish holdings with collective interests from private sector-friendly holdings only. Therefore, by using the term *social participation,* we do not distinguish the general popular participation from the participation of direct stakeholders.

In conclusion, Anvisa, Conitec, and ANS expanded, over the period analyzed, the provision of formal means of social participation. However, there is no clear policy or homogeneity of implementation and organization of patient and public involvement mechanisms among the institutions analyzed. We observed that the great number of participation occurs with public consultations, especially about medicines. Thus, the power of social participation to influence health technology decision making processes still needs thorough studies. In addition to identifying the stakeholders in these social participation structures, it is important to know who actually occupies them and whether this promotes more legitimate decisions.
