# Supplementary material for: Patient and public involvement in health technology decision-making processes in Brazil
Source: Rev Saude Publica. 2020 Nov 27;54:136. doi: 10.11606/s1518-8787.2020054002453 (PMC7702417; doi:10.11606/s1518-8787.2020054002453)
Supplement: Translation

# Translation: Participação social na gestão de tecnologias em saúde em âmbito federal no Brasil

**Authors:** Ana Carolina de Freitas Lopes, Hillegonda Maria Dutilh Novaes, Patricia Coelho de Soárez

**Affiliations:** I Brasil Universidade de São Paulo. Faculdade de Medicina. Programa de Pós-Graduação em Saúde Coletiva. São Paulo, SP, Brasil; II Brasil Universidade de São Paulo. Faculdade de Medicina. Departamento de Medicina Preventiva. São Paulo, SP, Brasil

**Keywords:** Participação da Comunidade, Participação Social, Participação dos Interessados, Tecnologia Biomédica, Avaliação da Tecnologia Biomédica, Agência Nacional de Vigilância Sanitária, Saúde Suplementar

## Abstract

**OBJETIVO:**

Caracterizar e discutir os processos formais de participação social previstos e implementados pelas instituições Agência Nacional de Vigilância Sanitária (Anvisa), Comissão Nacional de Incorporação de Tecnologias no SUS (Conitec) e Agência Nacional de Saúde Suplementar (ANS).

**MÉTODOS:**

Estudo exploratório, descritivo, comparativo, conduzido por meio de análise de documentos publicados até 2018 no sítio eletrônico e da legislação específica de cada instituição.

**RESULTADOS:**

Os espaços de participação social identificados incluem a representação em colegiados e participação direta por meio de uma demanda inicial ou em consultas públicas, audiências públicas e ouvidorias. A Anvisa conduziu 187 consultas públicas entre 1999 e 2018, e contabilizou 10.699 contribuições. Em 76 (41%) consultas públicas não há informações sobre as contribuições recebidas. A Conitec conduziu 234 consultas públicas, e recebeu 53.174 contribuições entre 2011 e 2018. Identificou-se que 70 (23%) recomendações da Conitec foram emitidas sem consulta pública, e 26 (8%) recomendações preliminares foram alteradas após consulta pública. As alterações de recomendação aconteceram especialmente em casos com grande volume de contribuições. A ANS conduziu 8 consultas públicas sobre o rol de procedimentos e eventos em saúde entre 2000 e 2018, e recebeu pelo menos 31.498 contribuições. Em três consultas públicas não há informações sobre o número de contribuições recebidas.

**CONCLUSÕES:**

Observa-se recente avanço normativo e de desempenho institucional em favor da participação social em espaços decisórios notadamente técnicos, embora de forma bastante heterogênea entre as instituições analisadas. O poder deliberativo da participação social de influenciar as decisões em saúde ainda necessita de estudo aprofundado, incluindo a caracterização dos participantes e a legitimidade das decisões subsequentes.

## INTRODUÇÃO

A institucionalidade da participação social, assim como a própria construção da democracia, está vinculada a contextos históricos específicos^1,2^. Em especial na América Latina, em que a instabilidade política e as desigualdades estruturais se mantêm constantes, tem sido desafiador propor e manter políticas voltadas para garantir a participação da sociedade nas instâncias públicas^1,2^.

A participação da sociedade nas decisões em saúde foi incluída como diretriz constitucional desde a criação do Sistema Único de Saúde (SUS), sendo regulamentada no arcabouço legal na forma de Conferências e Conselhos de Saúde, notadamente de forma descentralizada^3^. Nos anos seguintes, a participação social foi objeto de discussão e atuação política, tendo sido criados outros canais de interlocução entre governo e sociedade no âmbito da saúde^6^.

Paralelamente, a Política Nacional de Gestão de Tecnologias em Saúde (PNGTS), publicada em 2009, instituiu a racionalidade e o uso do conhecimento técnico e científico nas decisões coletivas em saúde, ainda que tendo como princípio o envolvimento dos diferentes atores da sociedade^7^. A gestão de tecnologias em saúde refere-se aos processos de avaliação, incorporação, difusão, gerenciamento da utilização e retirada de tecnologias no sistema de saúde^7^. Abrange, portanto, do registro sanitário de medicamentos, produtos e demais procedimentos em saúde às decisões de cobertura de planos de saúde e incorporações de tecnologias no SUS. Em âmbito federal, a gestão de tecnologias em saúde é conduzida pela Agência Nacional de Vigilância Sanitária (Anvisa), pela Comissão Nacional de Incorporação de Tecnologias no SUS (Conitec) e pela Agência Nacional de Saúde Suplementar (ANS).

A atuação da Anvisa inclui o registro inicial da tecnologia em saúde, da pesquisa clínica à autorização de uso, mediante comprovação científica de segurança e eficácia^8^. Para que uma tecnologia com autorização para produção e comercialização seja ofertada pelo SUS ou incluída no rol mínimo de procedimentos e eventos da saúde suplementar, exige-se análise adicional sobre segurança, eficácia, acurácia e efetividade, além de avaliação econômica comparativa de benefícios e custos com as tecnologias já disponibilizadas^4,9^. A decisão por incorporação, exclusão ou alteração de tecnologias no SUS é de competência do Ministério da Saúde (MS), assessorado pela Conitec^4^. Na saúde suplementar, a decisão cabe à Diretoria Colegiada da ANS, após consulta ao Comitê Permanente de Regulação da Atenção à Saúde (Cosaúde)^9^.

Em atenção às diretrizes do SUS e à legislação vigente, as instituições responsáveis pela gestão de tecnologias em saúde devem conciliar decisões técnicas e o envolvimento da sociedade. Iniciativas de envolvimento da sociedade conduzidas pela Anvisa, pela Conitec e pela ANS foram objeto de análises individuais em outros trabalhos^10^, mas ainda não de forma comparativa a partir do escopo da gestão de tecnologias em saúde. Este trabalho tem por objetivo caracterizar e discutir os espaços formais de participação social disponibilizados pelas instituições responsáveis pela gestão de tecnologias em saúde em âmbito federal no Brasil.

## MÉTODOS

Trata-se de estudo exploratório, descritivo, comparativo dos processos formais de participação social previstos e implementados pela Anvisa, pela Conitec e pela ANS. Para tanto, foi conduzida análise documental como método de coleta de dados, com consulta a documentos oficiais e legislação específica. A fonte primária de informações foi o sítio eletrônico das instituições, entendido como a principal ferramenta de acesso à informação disponível para a sociedade.

Foram incluídos documentos publicados no período entre a criação legal da instituição e dezembro de 2018. Assim, para a Anvisa, o período considerado foi entre 1999 e 2018; para a ANS, entre 2000 e 2018; e para a Conitec, de 2011 a 2018. Informações incompletas ou ausentes foram formalmente requisitadas às instituições por meio dos canais previstos na Lei de Acesso à Informação (Lei nº 12.527/2011)^15^.

Para análise dos dados, foi utilizado o método de análise descritiva. Informações sobre os espaços de participação social formalmente previstos e sobre consultas públicas já realizadas a respeito de tecnologias em saúde foram detalhadas para cada instituição. Privilegiou-se a apresentação das consultas públicas, dentre os demais espaços identificados, pela configuração de participação aberta, com comunicação direta entre os indivíduos e as instituições, sem a necessidade de representantes^16^. Além disso, sua realização é sempre prévia à tomada de decisão, com a prerrogativa de poder alterá-la.

De acordo com a PNGTS, são consideradas tecnologias em saúde os “medicamentos, equipamentos e procedimentos técnicos, sistemas organizacionais, informacionais, educacionais e de suporte, e programas e protocolos assistenciais por meio dos quais a atenção e os cuidados com a saúde são prestados à população”^7^.

Os espaços de participação social foram identificados como os processos por meio dos quais a sociedade pode expressar suas demandas. Como sociedade, foram consideradas todas as pessoas físicas ou jurídicas que não integram a estrutura governamental, incluindo usuários do SUS e dos planos de saúde, instituições e profissionais de saúde, setores da indústria e de serviços, além de organizações da sociedade civil.

Os processos de participação social foram classificados em cinco categorias: demandante inicial, representante em colegiados, consultas públicas, audiências públicas e ouvidorias. A atuação como demandante inicial é a prerrogativa da sociedade de iniciar o processo relacionado à gestão das tecnologias. Refere-se à solicitação de registro na Anvisa, de incorporação no SUS por meio da Conitec ou de inclusão no rol da ANS.

Representação em colegiados refere-se à participação de representantes da sociedade em instâncias colegiadas, criadas por iniciativa do poder público, a exemplo dos Conselhos de Saúde. Consultas e audiências públicas são mecanismos em que qualquer interessado pode contribuir, de forma escrita ou presencial. Ouvidorias públicas institucionais são instâncias de recebimento, encaminhamento e resolução de reclamações, solicitações e denúncias relativas a políticas e serviços públicos, abertas a qualquer cidadão^16^.

As consultas públicas realizadas por cada instituição foram categorizadas por tema e detalhadas quanto ao prazo para envio de contribuições e número de contribuições recebidas. Foram incluídas consultas públicas conduzidas por áreas da Anvisa relacionadas aos temas medicamentos, produtos para saúde e alimentos, por se enquadrarem no conceito de tecnologia em saúde apresentado na PNGTS. Foram excluídas as consultas públicas sobre agrotóxicos, cosméticos, saneantes, tabaco, sangue, tecidos e órgãos, e de gestão interna, por tratarem de temas não relacionados a tecnologias utilizadas na prestação direta de cuidados à saúde. Para inclusão, o prazo final para o envio de contribuições deveria ser até 31 de dezembro de 2018.

Para a Conitec, foram incluídas as consultas públicas sobre medicamentos, produtos ou procedimentos, com processos encerrados até 2018. Considerou-se como encerramento do processo a data da publicação da decisão pelo MS. As informações sobre as contribuições recebidas foram obtidas por meio da versão final dos relatórios técnicos disponíveis no sítio eletrônico da Conitec. Foram excluídas consultas públicas sobre Protocolos Clínicos e Diretrizes Terapêuticas (PCDT), uma vez que se referem a documentos de orientação de conduta clínica, organização dos serviços e implementação de tecnologias incorporadas em decisões prévias. O processo de elaboração dos PCDT, incluindo os mecanismos de envolvimento da sociedade, merece análise detalhada particular^14,17^.

Para a ANS, foram incluídas as consultas públicas sobre atualização do rol de procedimentos e eventos em saúde, portanto diretamente relacionadas a tecnologias em saúde, realizadas até 2018. Foram excluídas consultas públicas sobre temas alheios à gestão de tecnologias em saúde, como os assuntos de gestão financeira das operadoras de planos de saúde, da relação entre operadoras e prestadores de serviços ou de padronização de documentos.

Este trabalho compõe o projeto de pesquisa “Participação social na incorporação de tecnologias no SUS”, que obteve aprovação do Comitê de Ética em Pesquisa da Faculdade de Medicina da Universidade de São Paulo. Os documentos utilizados são de acesso público.

## RESULTADOS

Foi analisado um total de 606 documentos sobre o arcabouço legal das instituições e documentos de processos conduzidos por elas. Foram incluídos: 187 publicações de abertura de consulta pública no Diário Oficial da União e 99 relatórios de análise de participação social disponibilizados pela Anvisa; 304 relatórios técnicos publicados pela Conitec; oito termos de abertura de consultas públicas e 5 relatórios de consulta pública divulgados pela ANS; além das leis de criação de cada instituição (n = 3). Os espaços formais de participação social identificados nas instituições serão apresentados de forma comparativa, seguidos do detalhamento das consultas públicas realizadas.

### Espaços Formais de Participação Social

O Quadro apresenta os espaços formais de participação social por instituição. A solicitação à Anvisa de autorização para conduzir pesquisa clínica e para produção e comercialização de novos produtos está restrita ao produtor da tecnologia. Também cabe a ele a demanda por especificação do preço dos medicamentos e as alterações pós-registro. As decisões da agência sobre essas questões são embasadas exclusivamente em aspectos técnicos e processuais, sem abertura formal a participações externas, além do próprio demandante.

**Quadro:** Espaços formais de participação social na gestão de tecnologias em saúde em âmbito federal no Brasil, por instituição.

Instituição	Demandante inicial	Representação em colegiados	Consulta pública	Audiência pública	Ouvidoria
Anvisa	Produtor da tecnologia	**Conselho consultivo Permanente** Caráter consultivo e propositivo Representantes da sociedade (nº de indicados): CNI (1), CNC (1), comunidade científica (2), defesa do consumidor (2), CNS (1) e CNSaúde (1) **Câmaras setoriais** Caráter consultivo e de assessoramento Composição e duração fixadas caso a caso Deve incluir governo, setor produtivo e sociedade civil	Opcional	Opcional	Própria
Conitec	Qualquer pessoa (governo, setor produtivo ou sociedade civil)	**Plenário da Conitec** Permanente e deliberativo Representantes da sociedade (nº de indicados): CNS (1) e CFM (1)	Obrigatória	Opcional	Submetida à ouvidoria do Ministério da Saúde
ANS	Qualquer pessoa (governo, setor produtivo ou sociedade civil)	**Comitê Permanente de Regulação da Atenção à Saúde (Cosaúde)** Permanente e consultivo Representantes da sociedade (nº de indicados): CNS (1), profissionais de saúde (4), serviços de saúde (3), setor produtivo (2), sindicatos (3), empresas de saúde suplementar (6), defesa do consumidor (4), pacientes (2) **Câmaras técnicas** Discussões temáticas Composição e duração fixadas caso a caso	Opcional	**Opcional** Obrigatória apenas em caso de elaboração de anteprojeto de lei no âmbito da ANS	Própria

Anvisa: Agência Nacional de Vigilância Sanitária; Conitec: Comissão Nacional de Incorporação de Tecnologias no SUS; ANS: Agência Nacional de Saúde Suplementar; CNS: Conselho Nacional de Saúde; CNSaúde: Confederação Nacional de Saúde, Hospitais, Estabelecimentos e Serviços; CFM: Conselho Federal de Medicina; CNI: Confederação Nacional das Indústrias; CNC: Confederação Nacional do Comércio.

A representação em colegiados está prevista no conselho consultivo permanente da Anvisa e em câmaras setoriais temáticas temporárias, ambos de caráter consultivo. Dentre os 13 membros do conselho consultivo permanente, oito são representantes de instituições interessadas no âmbito de regulação da agência, e os demais são gestores federais, estaduais e municipais. Há previsão de condução de consultas e audiências públicas pela Anvisa, mas sem obrigatoriedade de realização. A Anvisa também conta com ouvidoria própria e com competências legalmente definidas.

De acordo com a norma, qualquer interessado pode atuar como demandante junto à Conitec e à ANS visando a incorporação no SUS ou na saúde suplementar. As restrições são de caráter técnico e processual. Dentre as exigências, destaca-se a apresentação sistematizada de evidências científicas sobre aspectos clínicos da tecnologia e de estudos específicos de avaliação econômica e impacto orçamentário.

As recomendações da Conitec quanto ao pedido de incorporação são proferidas por um Plenário, uma instância colegiada permanente e deliberativa. O caráter deliberativo do Plenário da Conitec refere-se à possibilidade de voto de seus membros em relação à recomendação final do pedido de incorporação, ainda que a decisão seja do MS. Dentre os 13 membros do Plenário, constam representantes do Conselho Nacional de Saúde (CNS) e do Conselho Federal de Medicina (CFM). Os demais são do próprio MS (incluindo todas as suas Secretarias, Anvisa e ANS) e gestores estaduais e municipais.

Consultas públicas sobre a incorporação de tecnologias no SUS são obrigatórias. As contribuições recebidas devem ser examinadas pelo Plenário antes da emissão da recomendação final. Há previsão para convocação de audiências públicas nos casos em que forem consideradas relevantes. A Conitec não conta com ouvidoria própria, mas está submetida à ouvidoria geral do MS, por meio da qual está sujeita à obrigatoriedade de resposta.

As propostas de inclusão no rol da ANS são discutidas no Cosaúde, um comitê permanente e consultivo. Entre os membros estão representantes de consumidores, operadoras, profissionais de saúde, sociedade civil organizada com interesse no setor e entidades governamentais. Consultas e audiências públicas são opcionais, sendo as audiências públicas obrigatórias apenas em caso de elaboração de anteprojeto de lei no âmbito da agência. A ouvidoria da ANS é específica e integra sua própria estrutura organizacional.

### Consultas Públicas

O comparativo temporal do número de consultas públicas realizadas sobre tecnologias em saúde pela Anvisa, pela Conitec e pela ANS até 2018 está apresentado no Figura 1.

**Figura 1:** Número de consultas públicas realizadas por ano, por instituição. Anvisa: Agência Nacional de Vigilância Sanitária; Conitec: Comissão Nacional de Incorporação de Tecnologias no SUS; ANS: Agência Nacional de Saúde Suplementar.

A Anvisa tem conduzido consultas públicas sobre temas relacionados a tecnologias em saúde desde 2008, com média e mediana de 17 consultas públicas por ano e um total de 187 consultas públicas realizadas entre 2008 e 2018.

A Conitec realizou em média 33 consultas públicas por ano entre 2011 e 2018 (mediana = 32), tendo acumulado um total de 234 consultas públicas no período. Observou-se que 70 recomendações da Conitec foram emitidas sem consulta pública, apesar de sua obrigatoriedade, representando 23% de todas as recomendações entre 2012 e 2018 (material suplementar). Dessas, 58 foram recomendações de incorporação e 12 de exclusão (desincorporação), sendo todas elas provenientes do setor público. A quase totalidade (n = 66, 94%) das recomendações sem consulta pública foram realizadas em demandas do próprio MS. As demais tinham como demandante a Anvisa, o Conasems, a Secretaria Estadual de Saúde de Pernambuco e o Poder Judiciário.

A ANS começou a conduzir consultas públicas sobre o rol de procedimentos e eventos em saúde em 2004, com frequência média bianual, de acordo com a periodicidade prevista para atualização do rol. Foram realizadas oito consultas públicas entre 2004 e 2018.

O comparativo do número de contribuições recebidas em consultas públicas está apresentado no Figura 2.

**Figura 2:** Número de contribuições em consultas públicas recebidas por ano, por instituição. Anvisa: Agência Nacional de Vigilância Sanitária; Conitec: Comissão Nacional de Incorporação de Tecnologias no SUS; ANS: Agência Nacional de Saúde Suplementar.

O volume de contribuições recebidas pela Anvisa foi de pelo menos 10.699. Não foram disponibilizadas informações sobre o resultado de 76 (41%) consultas públicas, todas com data anterior a 2014. O número de contribuições por consulta variou entre 2 e 3.531, com intervalo para contribuição entre 10 e 120 dias.

A Conitec recebeu 53.174 contribuições, uma média de 227 contribuições por consulta pública (mediana = 4.245). O tempo para contribuições variou entre 10 e 47 dias. Em 11 (5%) consultas públicas não foram recebidas sugestões. As contribuições recebidas apenas no ano de 2018 representam a metade de todas as contribuições já recebidas pela Conitec.

A ANS recebeu 31.498 contribuições nas consultas públicas realizadas sobre a atualização do rol entre 2009 e 2018. Não foram disponibilizadas informações sobre o volume de contribuições recebidas nas três consultas públicas realizadas antes de 2009. A média de contribuições recebidas é de 2.622 por consulta pública (mediana = 6.338), e o prazo para envio de contribuições variou entre 12 e 60 dias.

Em relação à divisão por temas, a Anvisa conduziu mais consultas públicas sobre medicamentos (n = 81, 43%), seguido por alimentos (n = 60, 32%) e produtos para saúde (n = 46, 25%). O volume de contribuições foi maior nas consultas sobre alimentos (n = 6.274, 59%). As consultas públicas sobre medicamentos receberam 3.012 contribuições (28%), e sobre produtos para saúde foram 1.413 (13%) contribuições.

Para a Conitec, as consultas públicas sobre medicamentos foram em maior número e tiveram maior participação da sociedade: 179 (76%) consultas públicas e 44.052 (83%) contribuições. Procedimentos foram pautados em 35 (15%) consultas e tiveram 1.870 (4%) contribuições. Produtos para saúde foram objeto de 20 (9%) consultas, nas quais foram recebidas 7.252 (14%) contribuições.

Identificou-se que 26 (8%) recomendações da Conitec foram alteradas após a consulta pública, principalmente de uma recomendação preliminar negativa para uma recomendação final favorável à incorporação. A maioria desses casos (73%) ocorreu nos anos 2017 e 2018 e teve como demandante a empresa produtora da tecnologia (62% dos casos). Aponta-se que, das dez consultas públicas com maior volume de contribuições recebidas pela Conitec, quatro tiveram a recomendação preliminar alterada após a consulta pública.

## DISCUSSÃO

Este trabalho apresentou os espaços de participação social disponibilizados pelas instituições brasileiras responsáveis pela gestão de tecnologias em saúde, em nível federal, na forma de representações em colegiados e da participação direta de interessados por meio de uma demanda inicial ou em consultas públicas, audiências públicas e ouvidorias. Anvisa, Conitec e ANS somaram 429 consultas públicas realizadas e 95.371 contribuições recebidas no período entre a criação legal da instituição e dezembro de 2018, apontando para a importância dada às consultas públicas como mecanismo de participação social nessas instituições.

Observou-se, por outro lado, que há heterogeneidade de funcionamento e implementação de espaços de participação social entre as instituições. As diferenças observadas neste trabalho referem-se ao caráter consultivo ou deliberativo dos colegiados, à qualificação e quantidade dos representantes, à vinculação ou discricionariedade de realização de consultas públicas e à disponibilização de informações sobre as consultas públicas realizadas e o impacto sobre as decisões finais.

Ainda que incluídas em uma política maior de gestão de tecnologias em saúde, observou-se que as três instituições apresentam escopo de atuação diverso, com diferentes objetos de análise e, por conseguinte, diferentes *stakeholders* e fontes de pressão política e social. Isso se reflete nas diferentes concepções de participação social observadas entre as instituições.

A Avaliação de Tecnologias em Saúde (ATS), que é a ferramenta utilizada nas três instituições para realizar suas avaliações, utiliza comumente o modelo de envolvimento do público proposto por Gauvin e colaboradores^18^. O modelo considera usuários do sistema de saúde, pacientes e profissionais de saúde como interessados no resultado da ATS. Esse modelo não inclui, pelo menos de forma explícita, atores-chaves na gestão de tecnologias em saúde no Brasil: produtores das tecnologias (como a indústria farmacêutica), operadoras de planos de saúde e o Poder Judiciário.

Neste trabalho, observou-se que a Conitec apresenta maior frequência de consultas públicas, devido à obrigatoriedade de realização em todos os processos de incorporação de tecnologias. No entanto, identificou-se que, a cada quatro recomendações da Conitec, uma é realizada sem consulta pública, especialmente em demandas do próprio Estado.

O tratamento diferenciado dado pela Conitec de acordo com a origem da demanda foi identificado em estudos prévios^19,20^. Caetano e colaboradores^19^ observaram que 32% das recomendações da Conitec sobre medicamentos até junho de 2016 foram proferidas sem consulta pública, incluindo de medicamentos sem registro na Anvisa, todos demandados pelo próprio MS. Yuba e colaboradoras^20^ observaram que demandas provenientes do setor público apresentavam maior frequência de recomendações favoráveis a incorporação, ainda que seus relatórios não apresentassem todos os requisitos formais de evidências clínicas e econômicas.

Neste trabalho sobre participação social, a consulta pública também recebeu tratamento diferenciado em demandas governamentais, tendo sido aprovada a proposta do demandante sem submetê-la a análise de forma pública, mesmo se tratando de tecnologias de grande relevância para a população atendida pelo SUS. Entre as tecnologias não submetidas a consulta pública, observou-se volume expressivo de recomendações de desincorporação, ou seja, de retirada de tecnologias que eram oferecidas pelo SUS. Entre esses casos estavam tecnologias em que há importante organização da sociedade civil, como a de pessoas que vivem com HIV e aids.

As alterações de recomendação sobre incorporação de tecnologias no SUS após consultas públicas, em especial nos casos com grande volume de contribuições, pode indicar uma permeabilidade institucional à intensidade da participação social, se considerarmos que o grande volume de contribuições implica sempre maior interesse da sociedade como um todo, o que exige, no entanto, análise dos segmentos que participaram das contribuições. Silva e colaboradores^14^ identificaram que seis recomendações da Conitec tiveram a recomendação preliminar alterada de “não incorporação” para “incorporação” entre 2012 e 2017. Em cinco delas houve nova proposta de preço por parte dos demandantes ou fato econômico relevante (disponibilidade de medicamento genérico) entre a recomendação preliminar e a recomendação final^14^. Essas observações indicam que a dimensão econômico-financeira é relevante nas decisões de incorporação de tecnologias no SUS, o que não é surpreendente.

Para a Anvisa e a ANS, destacam-se a ausência de obrigatoriedade de envolvimento do público e a substancial lacuna de informações sobre os processos de participação social desenvolvidos em seus anos iniciais de atuação, mesmo após requisição formal. Outros estudos também identificaram processos conduzidos pela Anvisa^10,12^, pela Conitec^14,21^ e pela ANS^13^ para envolver a sociedade em suas decisões. A ausência de espaços dialógicos e o caráter consultivo dos mecanismos disponíveis foram apontados como importantes limitadores de uma participação efetiva da sociedade na Anvisa e na ANS^10^. Para a Conitec, foram destacadas sua implementação recente, ausência de métodos sistemáticos e objetivos para análise das contribuições e ausência de avaliação de impacto das iniciativas implementadas^14,21^.

Mecanismos de participação social estão presentes em países cujo sistema de saúde e gestão de tecnologias são tomados como exemplos para o Brasil^22^. Os métodos utilizados são os mais diversos, prevalecendo a inclusão de representantes em conselhos e comitês^22,24,27,28^. A realização de consultas públicas abertas foi identificada na Inglaterra, no Canadá (Ontário), na Austrália e na Alemanha para o recebimento de comentários sobre relatórios e diretrizes clínicas nacionais^26,29,30^.

Aponta-se como limitação deste trabalho o fato de ter sido conduzida apenas uma mensuração quantitativa da participação da sociedade nos espaços decisórios, e não uma avaliação da efetividade da participação social promovida por essas organizações. Assim, coloca-se como importante perspectiva a avaliação da forma como os gestores incluem as contribuições da sociedade em seus processos decisórios, se o fazem de fato, a partir das demandas de quais interessados e se promovem a participação social equitativa.

Outra limitação importante é que não foram identificados os participantes desses espaços. Na definição de sociedade como qualquer pessoa física ou jurídica não integrante da estrutura governamental, igualaram-se, para fins deste trabalho, empresas, profissionais, consumidores, movimentos sociais e indivíduos isolados. Como instituições de regulação de um setor com grandes interesses de mercado, seria imperativo distinguir participações com interesses coletivos das participações favoráveis unicamente ao setor privado. Ao utilizar o termo *participação social*, não distinguimos, portanto, a participação popular em geral da participação de interessados diretos.

Por fim, conclui-se que Anvisa, Conitec e ANS ampliaram, ao longo do período analisado, a disponibilização de espaços formais de participação social. Não se observam, entretanto, uma política clara ou homogeneidade de implementação e organização desses espaços entre as instituições analisadas. Observou-se que o maior volume de participação ocorre por meio de consultas públicas, em especial sobre medicamentos. Coloca-se em perspectiva que o poder deliberativo da participação social de influenciar as decisões em saúde ainda necessita de estudo aprofundado. Além de identificar a presença da sociedade nesses canais, importa conhecer quem de fato os ocupa e se isso promove decisões mais legítimas.
